# Development and validation of UPLC-MS/MS method for icariin and its metabolites in mouse urine

**DOI:** 10.3389/fphar.2024.1389754

**Published:** 2024-06-11

**Authors:** Na Li, Mei Yuan, Jinjing Che

**Affiliations:** State Key Laboratory of Toxicology and Medical Countermeasures, Beijing Institute of Pharmacology and Toxicology, Beijing, China

**Keywords:** icariin, metabolite, UPLC-MS/MS, pharmacokinetics, urinary drug concentration, urine sample testing

## Abstract

An ultra-performance liquid chromatography-tandem mass spectrometry (UPLC-MS/MS) method was utilized to develop a technique for the simultaneous quantification of icariin and its primary metabolites in mouse urine. The levels of icariin, icariside Ⅰ, icariside Ⅱ, baohuoside Ⅱ, wushanicaritin, icaritin, and desmethylicaritin in mouse urine were analyzed subsequent to the oral administration of an icariin suspension. This study aimed to preliminarily investigate the excretion profile of icariin in mice. Using an aqueous solution containing 0.1% formic acid (A) and an acetonitrile solution containing 0.1% formic acid (B) as the mobile phases, icariin and its major metabolites demonstrated satisfactory linearity over the concentration range of 0.25–800 ng·mL^−1^. The precision and accuracy of intra-day and inter-day measurements were all found to be within 15%. Seventy-two hours after the intragastric administration of icariin suspension to a mouse, the cumulative urinary excretion of icariin, icariside Ⅰ, icariside Ⅱ, baohuoside Ⅱ, wushanicaritin, icaritin, and desmethylicaritin was quantified as 13.48, 18.70, 2,627.51, 2.04, 10.04, 3,420.44, and 735.13 ng, respectively. The UPLC-MS/MS method developed in this research is characterized by its simplicity, sensitivity, and speed, making it well-suited for the concurrent quantification of icariin and its associated metabolites in urine. Additionally, it is appropriate for analyzing urine samples that may contain multiple drugs in future investigations.

## Graphical Abstract

**Figure:**
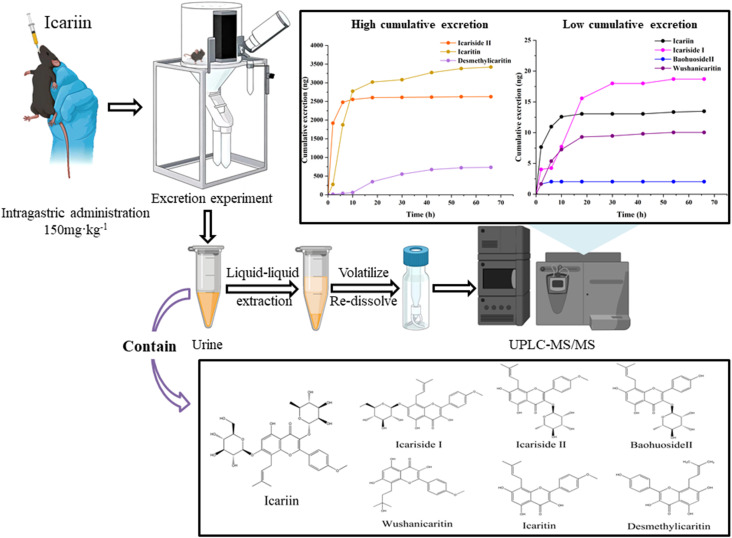
Effect of rGO doping in Tellurium tubes to achieve high electrochemical performance.

## 1 Introduction

A growing body of research has indicated that icariin serves as the primary bioactive constituent in Epimedium brevicornu Maxim, a traditional Chinese herb with a long history of use (Xu et al., 2017). Icariin is a flavonoid compound that is isoprenylated, characterized by the molecular formula C_33_H_40_O_15_ and a molecular weight of 676.67 g·moL^−1^. It comprises a glucosyl group at C-3, a methoxy group at C-4, a rhamnosyl group at C-7, and an isopentenyl group at C-8 (Su et al., 2023). It represents the most prevalent component in Epimedium and has been identified as the exclusive chemical marker for assessing the quality of flavonoids according to the latest Chinese Pharmacopoeia (Chinese Pharmacopoeia Commission, 2020a). It exhibits pharmacological effects in diverse areas, including neurodegenerative diseases, cardiovascular disorders, anti-osteoporosis, anti-inflammatory properties, enhancement of the reproductive system, anti-oxidative stress, anti-depressant effects, anti-tumor properties, among others (He et al., 2020).

With the increasing interest in icariin, research on its *in vivo* mechanisms is expanding. Various methodologies have been employed to explore the pharmacokinetic properties of icariin and its metabolites like capillary zone electrophoresis (CZE), gas chromatography, and liquid chromatography (Shen et al., 2007; Chen et al., 2009; Lu et al., 2015). Xu et al. used LC-MS/MS to detect icariin in rat plasma and tissues, including the liver, heart, spleen, lung, kidney, brain, testicle, uterus, and ovary, achieving a limit of detection of 1 ng‧mL^−1^ and a linearity range of 1–500 ng‧mL^−1^ (Xu et al., 2017). Sun et al. employed HPLC-MS/MS to identify icariin, baohuoside Ⅰ, and baohuoside Ⅱ in rat plasma, with detection limits of 4, 2, and 1 ng‧mL^−1^, respectively, and linearity ranges of 4–800, 2–400, and 1–200 ng‧mL^−1^, respectively (Sun et al., 2018b). Liu et al. utilized CZE to detect icariin, icaritin, and desmethylicaritin in rat serum. The minimum detectable concentration of icariin was found to be 1 mg‧L^−1^, with a linearity range of 2.5–150 mg‧L^−1^ (Liu and Lou, 2004). However, these approaches have limitations, including the utilization of a large quantity of samples, dependence on time-consuming and sample-consuming derivatization procedures, lack of sufficient sensitivity, or the analysis of only one or two of its metabolites (Shen et al., 2009).

**FIGURE 1:**
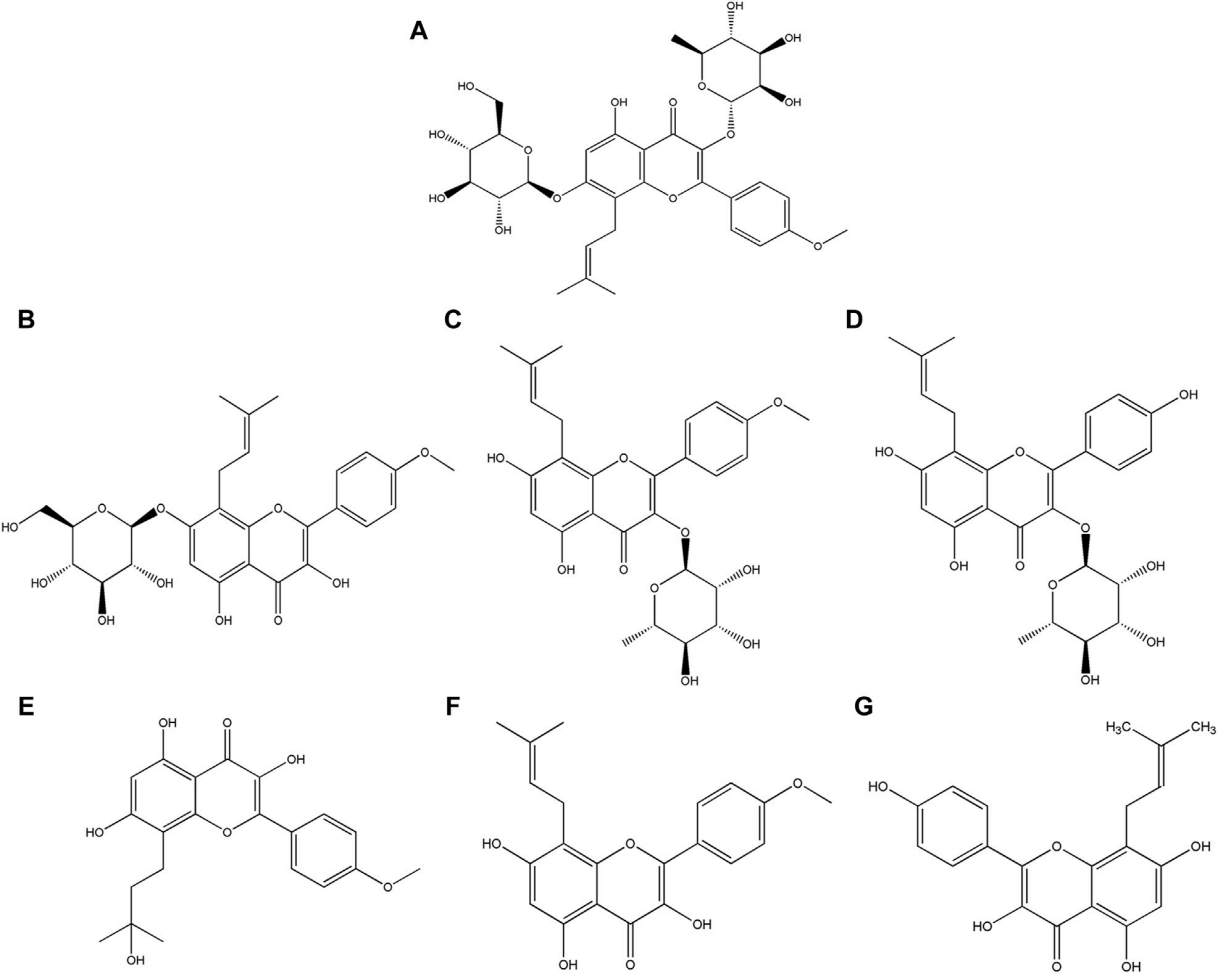
Structural formula of **(A)** Icariin, **(B)** Icariside Ⅰ, **(C)** Icariside Ⅱ, **(D)** Baohuoside Ⅱ, **(E)** Wushanicaritin, **(F)** Icaritin, and **(G)** Desmethylicaritin.

Yu et al. conducted a study showing that the unaltered structure of icariin was minimally present in urine, consisting less than 0.43‰. This suggested that the majority of icariin underwent predominant metabolism into various metabolites prior to excretion. It is imperative to conduct an investigation into the *in vivo* drug disposition of icariin and to characterize its metabolites. This information is crucial for comprehending the biological activity of icariin (Yu et al., 2016). Icariin undergoes a multifaceted transformation process *in vivo*, such as deglycosylation, demethylation, dehydrogenation, hydroxylation, epoxidation, reduction, oxidation, and conjugation with glucuronide (Qian et al., 2012; Bi et al., 2022). A variety of metabolites have been identified, including icariside Ⅰ, icariside Ⅱ, baohuoside Ⅱ, wushanicaritin, icaritin, and desmethylicaritin, with their structural formulas illustrated in Figure 1 (Xu et al., 2007; Shen et al., 2009). The removal of rhamnose or glucose residues from icariin leads to the formation of icariside Ⅰ or icariside Ⅱ, respectively. Icariside Ⅱ has the potential to undergo demethylation to form baohuoside Ⅱ. Icaritin represents the aglycone form of icariin. Icaritin can be demethylated to desmethylicaritin and oxidized to wushanicaritin (Chen et al., 2011). The active substances that mediate the medicinal effects of traditional Chinese medicines are typically prototypical components, metabolized components, or modified endogenous components that enter the bloodstream (Ma et al., 2024). It has been reported that the main metabolites icariside I, icariside II, and icaritin exhibit comparable biological activities, such as antidiabetic, anti-osteoporosis, anticancer, and neuroprotective effects, to icariin (Sun et al., 2016; Kim et al., 2017; Yan et al., 2017; Sun et al., 2018a; Chen et al., 2018). He et al. have found that icariside II demonstrates significantly greater central pharmacological effectiveness than icariin when administered peripherally (He et al., 2020). Therefore, it is imperative that pharmacokinetic studies of icariin prioritize the investigation of its metabolites to elucidate the disparity observed between bioassay results and pharmacological effects (Wu et al., 2016).

Drugs that are introduced into the body eventually undergo an excretory process for elimination from the body. Excretion studies serve the dual purpose of elucidating the elimination process of the parent drug from the body and are intricately linked to the preservation of drug efficacy. Therefore, it is essential to investigate the metabolites present in urine (Zhang and Zhang, 2017). A simple, sensitive, and dependable technique is required to simultaneously measure icariin and its metabolites in limited urine samples. This is crucial for a thorough comprehension of the pharmacokinetic disposition of bioactive compounds after the oral intake of icariin. To date, there has not been a singular assay available for the simultaneous quantification of the seven active components specified in urine samples. In this investigation, a UPLC-MS/MS method was formulated and authenticated for the concurrent quantification of icariin and its six derivatives. The method employed liquid-liquid extraction, eliminating the need for intricate sample preparation steps like derivatization. This validated method was rapid, simple, and sensitive, enabling the accurate detection and quantification of all seven analytes in mouse urine with high precision and accuracy after the oral administration of icariin. These analytes included icariin, icariside Ⅰ, icariside Ⅱ, baohuoside Ⅱ, wushanicaritin, icaritin, and desmethylicaritin. Moreover, this study conducted a concise analysis of the urinary excretion mechanism of icariin and its associated metabolites in C57 mice. C57 mice participated in this experiment as background mice of APP/PS1 transgenic mice. This analysis offered a foundational approach for understanding the urinary excretion profile and facilitating the pharmacokinetic studies of icariin.

## 2 Materials and methods

### 2.1 Chemicals and reagents

Icariin (purity ≥98%) was purchased from the National Institute for Food and Drug Control (Beijing, China). Icariside Ⅰ (purity ≥98%), icariside Ⅱ (purity ≥98%), and wushanicaritin (purity ≥98%) were purchased from OKA Bio-Technology Co., Ltd. (Beijing, China). Baohuoside Ⅱ (purity of 98%) was purchased from BioBioPha Bio-Technology Co., Ltd. (Kunming, China). Icaritin (purity ≥98%) was purchased from Meilune Bio-Technology Co., Ltd. (Dalian, China). Desmethylicaritin (purity ≥97%) was purchased from Yuanye Bio-Technology Co., Ltd. (Shanghai, China). Propranolol (purity >99%) was purchased from U.S. Pharmacopeia Standard R&D and Technical Service (Shanghai) Co., Ltd. (Shanghai, China). Dimethyl sulfoxide (purity of 99.7%) was purchased from Innochem Science and Technology Co., Ltd. (Beijing, China). HPLC-grade methanol, HPLC-grade acetonitrile, and HPLC-grade formic acid were purchased from Thermo Fisher Scientific (CHINA) Co., Ltd. (Shanghai, China). Chemical-grade sodium carboxymethyl cellulose was purchased from Sinopharm Chemical Reagent Co., Ltd. (Shanghai, China). All other chemicals and reagents used in this study were of analytical grade and were commercially available.

### 2.2 Animals

SPF-grade male C57 mice weighing 20–22 g each were purchased from Vital River Laboratory Animal Technology Co., Ltd. (Beijing, China) (laboratory animal production license number: SCXK (Beijing) 2021-0006). The animal study was reviewed and approved by the Animal Ethics Committee of the Beijing Institute of Pharmacology and Toxicology (animal ethics number: IACIC-DWZX-2021-763). Acclimatization feeding was performed over a period of 3 days, with a 12-h day/night cycle. The temperature and the relative humidity levels in the laboratory were 22°C–24°C and 40%–60%, respectively. Prior to drug administration, the mice were fasted for 12 h with free access to water. All experiments involving animals adhered to the Guidelines for the Care and Use of Laboratory Animals.

### 2.3 UPLC-MS/MS instrumentation and analytical conditions

Biological samples were analyzed using a UPLC-MS/MS system (Agilent 1290-6410, United States). Chromatographic separation was achieved using a Shiseido CAPCELL PAK MGⅡ C18 column (2.0 mm × 100 mm, 3.0 μm) at a controlled temperature of 45°C. The mobile phases consisted of an aqueous solution containing 0.1% formic acid (A) and an acetonitrile solution containing 0.1% formic acid (B), utilizing a gradient elution program with the following conditions: 0.00–4.50 min, 25%–60% B; 4.50–6.00 min, 60%–90% B; 6.00–6.50 min, 90%–25% B. Each sample underwent analysis for 7 min. The flow rate was adjusted to 0.4 mL‧min^−1^ and the injection volume was 10 μL.

Analyses were performed using an electrospray ionization (ESI) source in positive ion mode multiple reaction monitoring (MRM). The mass spectrometry parameters were meticulously optimized, including a nebulizer pressure of 30 psi, an ion source temperature of 350°C, a capillary voltage of 4000 V, and a nitrogen gas flow rate of 10 L·min^−1^. The precursor-to-product ion transitions monitored for icariin, icariside Ⅰ, icariside Ⅱ, baohuoside Ⅱ, wushanicaritin, icaritin, desmethylicaritin, and propranolol (the internal standard) were as follows: m/z 677.3→369.2, 531.3→369.2, 514.9→369.1, 501.0→355.0, 387.0→313.0, 369.0→313.0, 355.1→299.0, and 260.0→116.2, respectively. The corresponding fragmentor voltages were set at 142, 137, 88, 94, 122, 120, 116, and 120 V, and the collision energies were adjusted to 28, 16, 8, 7, 28, 28, 27, and 16 eV.

### 2.4 Solution preparation

Icariin is characterized as a lipophilic, pale yellow, and acicular crystalline powder. The powder of icariin was precisely weighed and then dispersed in a solution of 0.5% sodium carboxymethyl cellulose (CMC-Na). Following ultrasonication, the icariin suspension with a concentration of 9 mg·mL^−1^ was prepared, and the dosage for oral administration in mice was 150 mg·kg^-1^.

### 2.5 Preparation of standard series samples and quality control (QC) samples

Appropriate quantities of icariin, icariside Ⅰ, icariside Ⅱ, baohuoside Ⅱ, wushanicaritin, icaritin, and desmethylicaritin standards were accurately weighed and dissolved in dimethyl sulfoxide (DMSO) to prepare stock solutions with a concentration of 2 mg·mL^−1^. Similarly, a precise quantity of propranolol standard was weighed and dissolved in DMSO to create an internal standard solution at a concentration of 2 mg·mL^−1^. These solutions were subsequently stored at 4°C for stability. A calibration curve was established by using the internal standard solution of propranolol at a concentration of 100 ng·mL^−1^ and a range of standard urine samples at nine different concentrations, specifically 0.25, 1, 2, 5, 50, 100, 200, 400, and 800 ng·mL^−1^. Furthermore, samples were prepared for quality control at three different concentrations of 0.5, 20, and 600 ng·mL^−1^ to verify the accuracy and precision of the assay.

### 2.6 Urine samples preparation

100 μL of urine was transferred into a 1.5 mL centrifuge tube, followed by the addition of 5 μL of a propranolol internal standard solution with a concentration of 100 ng·mL^−1^. Following vortex-mixing, 500 μL of an ethyl acetate solution was added for liquid-liquid extraction. The mixture was vortex-mixed again at 3,000 rpm for 1 min and subsequently centrifuged at 4°C, 16,000×*g* for 15 min. The supernatant was collected carefully and evaporated to dryness at 45°C. The dried residue was re-dissolved in 50 μL of a solution containing 60% methanol in water. The mixture was vortexed for 1 min to ensure thorough mixing and then centrifuged at 4°C, 16,000×*g* for 15 min. Finally, 10 μL of the resulting clear supernatant was injected into the UPLC-MS/MS system for subsequent analysis.

### 2.7 Bioanalytical method validation

A comprehensive validation of the established bioanalytical method was performed according to the guidelines outlined in the “9012 Guidelines for Quantitative Bioanalytical Method Validation” of the Chinese Pharmacopoeia (Chinese Pharmacopoeia Commission, 2020b). The essential validation tests for the assay encompass selectivity, calibration curve and lower limit of quantification (LLOQ), precision and accuracy, extraction recovery and matrix effect, stability, and dilution integrity.

#### 2.7.1 Selectivity

100 μL of blank urine was collected, with the internal standard solution being replaced by methanol. The sample was processed in accordance with the protocol outlined in Section 2.6. The chromatograms were subsequently recorded. A 100 μL urine sample with a concentration of 0.25 ng·mL^−1^ was obtained and processed following the methodology described in Section 2.6, and the chromatograms were then recorded. A 100 μL urine sample was collected from C57 mice within 0–4 h after the intragastric administration of icariin suspension at a dosage of 150 mg kg^-1^. The sample was processed according to the protocol specified in Section 2.6, and the resulting chromatograms were documented. The selectivity of the method was evaluated by comparing these individual chromatograms.

#### 2.7.2 Calibration curve and lower limit of quantification

100 μL of urine samples at different concentrations (0.25, 1, 2, 5, 50, 100, 200, 400, and 800 ng·mL^−1^) were prepared and processed according to the protocol described in Section 2.6 before undergoing analysis. Five replicates were prepared for each concentration level. Plotted on the horizontal axis (X) was the concentration of each analyte in the urine, while the vertical axis (Y) represented the ratio of the peak area of the analyte to the peak area of the internal standard. Conducted linear regression using the 1/X^2^ weighted least squares method. Determined the LLOQ based on a signal-to-noise ratio (S/N) of 5. Subsequently, calibration curves, correlation coefficients, and linear ranges were derived for icariin, icariside I, icariside II, baohuoside II, wushanicaritin, icaritin, and desmethylicaritin. The recalculated concentrations of the calibration standards were typically expected to fall within ±15% of the nominal value, with the exception of the LLOQ, where they should be within ±20% of the nominal value.

#### 2.7.3 Precision and accuracy

Quality control samples (*n* = 5) at the LLOQ (0.25 ng·mL^−1^) and at three additional concentrations (0.5, 20, and 600 ng·mL^−1^) were prepared and processed according to the protocol described in Section 2.6 over three consecutive days. The concentrations of the quality control samples were determined by using the respective daily calibration curves to evaluate the precision and accuracy of the method. Intra-day and inter-day variability were assessed using the relative standard deviation (RSD) and relative error (RE). For the quality control samples, the acceptance criteria for precision and accuracy were set at ±15% of the nominal values, except the LLOQ, which had an acceptable deviation of ±20% from the nominal value.

#### 2.7.4 Extraction recovery and matrix effect

Quality control samples were prepared at low, medium, and high concentrations (0.5, 20, 600 ng·mL^−1^) and processed according to the protocol described in Section 2.6 to acquire peak areas, denoted as S_A_. Concurrently, blank urine samples were dried using the same protocol, followed by re-dissolving the dried residue with standard solutions at low, medium, and high concentrations (prepared in 60% methanol). The resulting peak areas were denoted as S_B_. Similarly, standard solutions at the three concentrations above (also prepared in 60% methanol) were directly analyzed to determine peak areas, denoted as S_C_. Five replicates were prepared for each concentration level. The quantification of extraction recovery was conducted by calculating the S_A_/S_B_ ratio, while the assessment of matrix effect was performed by determining the S_B_/S_C_ ratio.

#### 2.7.5 Stability

Low-quality control (LQC) samples at 0.5 ng·mL^−1^, medium-quality control (MQC) samples at 20 ng·mL^−1^, and high-quality control (HQC) samples at 600 ng·mL^−1^ (*n* = 5) were prepared for stability assessments. These assessments included storage for 6 h at room temperature (25°C), 24 h in the autosampler at 4°C, long-term storage at −20°C for 7 days, and three freeze-thaw cycles at −20°C. Following the aforementioned stability conditions, the QC samples were processed following the protocol detailed in Section 2.6. The stability of the analytes under these conditions was assessed by calculating the RE based on the variance between the measured values and the theoretical values.

#### 2.7.6 Dilution integrity

Urine samples were prepared by diluting high-concentration samples containing icariside II, icaritin, and desmethylicaritin to a concentration of 600 ng·mL^−1^ with blank urine at a 20-fold ratio (v/v). Five replicates were prepared in parallel and treated following the procedure outlined in Section 2.6. The concentrations of the samples were calculated based on the respective daily calibration curves to assess the precision and accuracy of the measured concentrations.

### 2.8 Application to urine sample testing

A male C57 mouse was randomly selected and subjected to a 12-h fasting period while having unrestricted access to water. The mouse was then housed in a metabolic cage and orally administered an icariin suspension at a dosage of 150 mg·kg^-1^. Subsequent urine samples were collected at specific time intervals both pre- and post-administration (0–4, 4–8, 8–12, 12–24, 24–36, 36–48, 48–60, and 60–72 h). The volume of urine gathered in each time interval was carefully recorded and preserved at −20°C until subsequent processing. During the urine collection period, the mouse had continuous access to food and water to support its regular physiological metabolism.

### 2.9 Data collection and analysis

The experimental data were expressed as mean ± standard deviation (SD) and analyzed using Excel (version 2010, Microsoft Corporation, United States).

## 3 Results

### 3.1 Bioanalytical method validation

#### 3.1.1 Selectivity

**FIGURE 2:**
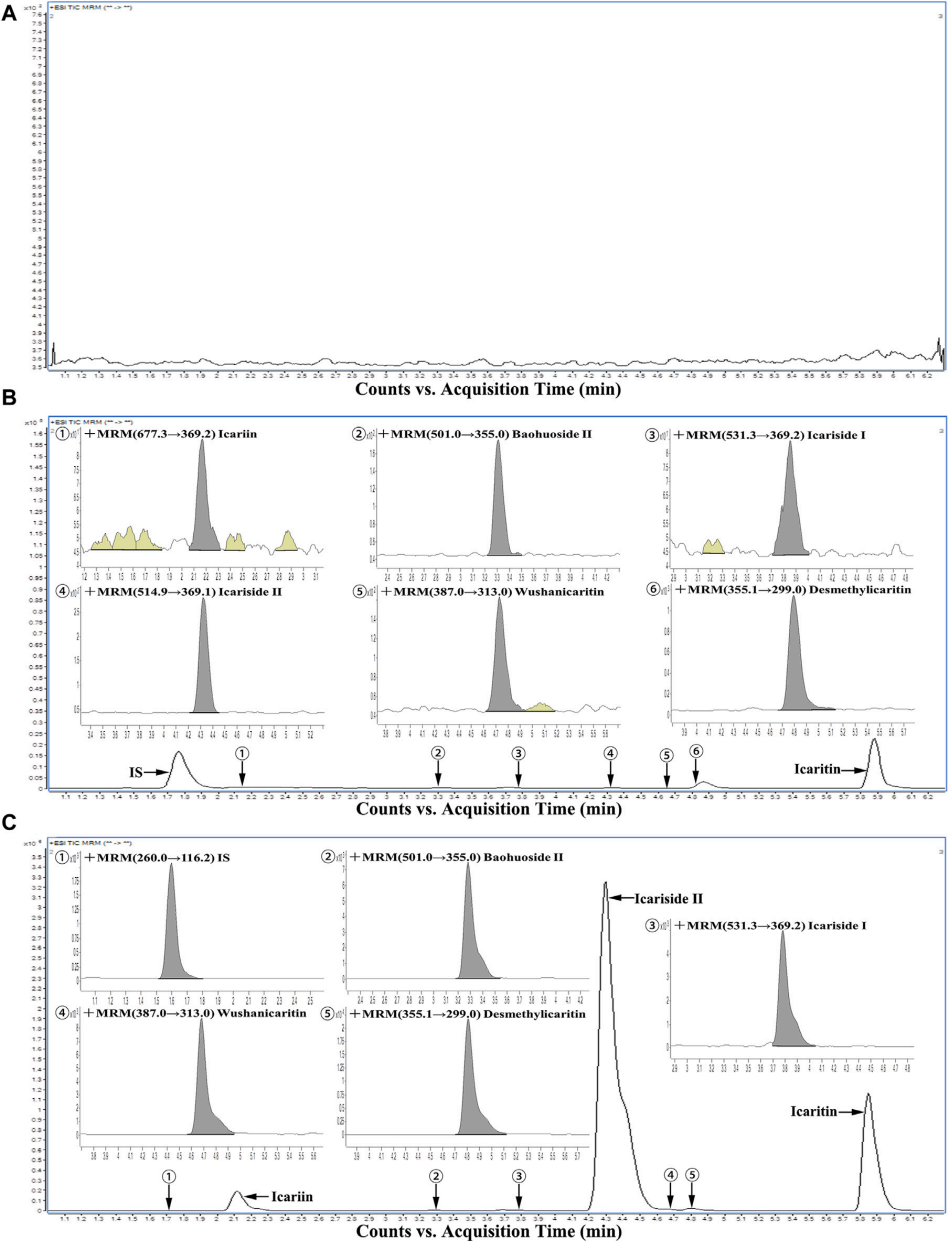
Representative chromatograms of all analytes in urine samples: **(A)** A blank urine sample; **(B)** A LLOQ sample; **(C)** A urine sample during 0–4 h after intragastric administration of icariin suspension at a dosage of 150 mg·kg^−1^.

As illustrated in Figure 2, under the specified analytical conditions, the retention times for icariin, icariside Ⅰ, icariside Ⅱ, baohuoside Ⅱ, wushanicaritin, icaritin, desmethylicaritin, and the internal standard were 2.16, 3.86, 4.30, 3.29, 4.67, 5.86, 4.81, and 1.71 min, respectively. The peak shapes of each analyte intended for measurement exhibited satisfactory characteristics, and the presence of endogenous constituents in the biological matrix did not interfere with the quantification of icariin and its associated metabolites, demonstrating the selectivity of the method.

#### 3.1.2 Calibration curve and LLOQ

As demonstrated in Table 1, icariin and its associated metabolites displayed good linearity over the range of 0.25–800 ng·mL^−1^ (*R* ^2^ > 0.99). The lower limit of quantification, which was determined as the minimum concentration point on the standard curve, exhibiting precision and accuracy results within ±15%, was set at 0.25 ng·mL^−1^. The aforementioned limits fulfilled the criteria for content analysis and proved to be satisfactory for the urinary excretion study of icariin, icariside Ⅰ, icariside Ⅱ, baohuoside Ⅱ, wushanicaritin, icaritin, and desmethylicaritin in mice following the oral administration of icariin suspension at a dosage of 150 mg·kg^−1^.

**TABLE 1:** Linearity of icariin, icariside Ⅰ, icariside Ⅱ, baohuoside Ⅱ, wushanicaritin, icaritin, and desmethylicaritin in mouse urine (*n* = 3).

Analyte	Batch	Regression equation	*R* ^2^	Linear range (ng·mL^−1^)	LLOQ (ng·mL^−1^)
Icariin	1	y = 2.7772x + z 0.0191	0.9991	0.25–800	0.25
	2	y = 2.7565x + 0.0188	0.9909		
	3	y = 2.8291x + 0.0219	0.9928		
Icariside Ⅰ	1	y = 5.0696x + 0.0241	0.9997		
	2	y = 5.5787x + 0.0078	0.9976		
	3	y = 5.2108x + 0.0083	0.9985		
Icariside Ⅱ	1	y = 14.1864x + 0.3376	0.9965		
	2	y = 14.5164x + 0.2577	0.9925		
	3	y = 16.3200x + 0.2416	0.9957		
Baohuoside Ⅱ	1	y = 17.2912x − 0.0166	0.9952		
	2	y = 17.5009x − 0.0033	0.9958		
	3	y = 19.3972x − 0.0316	0.9926		
Wushanicaritin	1	y = 19.7794x − 0.0046	0.9929		
	2	y = 19.8069x + 0.0035	0.9959		
	3	y = 19.4818x − 0.0173	0.9929		
Icaritin	1	y = 31.0888x + 0.2894	0.9942		
	2	y = 26.1483x + 0.2789	0.9947		
	3	y = 18.4033x + 0.6171	0.9912		
Desmethylicaritin	1	y = 9.6121x + 0.0642	0.9912		
	2	y = 9.7417x + 0.0296	0.9949		
	3	y = 9.2088x + 0.0130	0.9953		

#### 3.1.3 Precision and accuracy

As indicated in Table 2, the intra-day precision ranges of icariin, icariside Ⅰ, icariside Ⅱ, baohuoside Ⅱ, wushanicaritin, icaritin, and desmethylicaritin at low, medium, and high quality control concentrations (0.5, 20, and 600 ng·mL^−1^) were 2.09%–7.60%, 3.95%–9.09%, 2.40%–9.90%, 3.82%–7.60%, 3.00%–6.89%, 3.84%–8.09%, and 5.04%–8.37%, respectively. The inter-day precision ranges of the same compounds were 4.38%–7.69%, 5.31%–11.54%, 4.80%–8.33%, 5.60%–11.11%, 4.52%–12.50%, 3.85%–12.64%, and 5.62%–12.50%, respectively. The intra-day accuracy ranges were as follows: −2.67%–8.16%, −5.35%-2.67%, −5.33%-2.00%, −4.40%–6.67%, −4.00%-1.16%, −2.48%–5.33%, and −4.00%-0.78%. The inter-day accuracy ranges were −2.50%–8.16%, −5.35%-4.00%, −4.00%-2.00%, −4.40%–8.00%, −4.00%-1.16%, −2.48%–4.00%, and −4.00%-0.77%. The results demonstrated good precision and accuracy for each analyte, suggesting that the assay was accurate and reliable, meeting the requirements for *in vivo* sample analysis.

**TABLE 2:** Intra-day and inter-day precision and accuracy of UPLC-MS/MS analysis of icariin, icariside Ⅰ, icariside Ⅱ, baohuoside Ⅱ, wushanicaritin, icaritin, and desmethylicaritin in mouse urine (Mean ± SD, *n* = 5).

Analyte	Concentration (ng·mL^−1^)		RSD (%)		RE (%)	
	Expected	Measured	Intra-day (*n* = 5)	Inter-day (*n* = 15)	Intra-day (*n* = 5)	Inter-day (*n* = 15)
Icariin	0.25 (LLOQ)	0.26 ± 0.02	7.60	7.69	5.33	4.00
	0.5 (LQC)	0.49 ± 0.03	7.54	6.12	−2.67	−2.00
	20 (MQC)	19.50 ± 1.58	5.87	8.10	−2.50	−2.50
	600(HQC)	648.94 ± 28.45	2.09	4.38	8.16	8.16
Icariside Ⅰ	0.25 (LLOQ)	0.26 ± 0.03	9.09	11.54	2.67	4.00
	0.5 (LQC)	0.51 ± 0.04	6.58	7.84	1.33	2.00
	20 (MQC)	19.03 ± 1.01	5.15	5.31	−4.82	−4.85
	600(HQC)	567.91 ± 39.36	3.95	6.93	−5.35	−5.35
Icariside Ⅱ	0.25 (LLOQ)	0.24 ± 0.02	9.90	8.33	−5.33	−4.00
	0.5 (LQC)	0.51 ± 0.03	5.88	5.88	2.00	2.00
	20 (MQC)	19.80 ± 0.95	3.91	4.80	−1.02	−1.00
	600(HQC)	606.08 ± 30.31	2.40	5.00	1.01	1.01
Baohuoside Ⅱ	0.25 (LLOQ)	0.27 ± 0.03	7.60	11.11	6.67	8.00
	0.5 (LQC)	0.49 ± 0.03	6.78	6.12	−1.33	−2.00
	20 (MQC)	19.12 ± 1.07	5.20	5.60	−4.40	−4.40
	600(HQC)	591.87 ± 47.77	3.82	8.07	−1.36	−1.36
Wushanicaritin	0.25 (LLOQ)	0.24 ± 0.03	6.89	12.50	−2.67	−4.00
	0.5 (LQC)	0.48 ± 0.03	5.54	6.25	−4.00	−4.00
	20 (MQC)	19.48 ± 0.88	4.01	4.52	−2.60	−2.60
	600(HQC)	606.96 ± 36.55	3.00	6.02	1.16	1.16
Icaritin	0.25 (LLOQ)	0.26 ± 0.01	5.08	3.85	5.33	4.00
	0.5 (LQC)	0.52 ± 0.02	3.84	3.85	4.67	4.00
	20 (MQC)	20.62 ± 2.14	8.09	10.38	3.08	3.10
	600(HQC)	585.15 ± 73.98	6.99	12.64	−2.48	−2.48
Desmethylicaritin	0.25 (LLOQ)	0.24 ± 0.03	8.37	12.50	−4.00	−4.00
	0.5 (LQC)	0.49 ± 0.04	8.16	8.16	−2.00	−2.00
	20 (MQC)	20.09 ± 1.13	5.04	5.62	0.47	0.45
	600(HQC)	604.65 ± 57.26	6.75	9.47	0.78	0.77

#### 3.1.4 Extraction recovery and matrix effect

In this study, icariin, icariside Ⅰ, icariside Ⅱ, baohuoside Ⅱ, wushanicaritin, icaritin, and desmethylicaritin were extracted from urine samples using the ethyl acetate liquid-liquid extraction technique. Table 3 presents the outcomes of extraction recoveries and matrix effects of icariin and its associated metabolites at low, medium, and high concentrations of quality control in urine samples. The mean extraction recoveries ranged from 82.21% to 92.32%, 78.04%–91.46%, 96.81%–108.71%, 83.48%–92.02%, 100.18%–110.16%, 104.13%–118.54%, and 99.70%–110.97%, respectively. The mean matrix effects ranged from 84.74% to 89.50%, 98.47%–108.14%, 108.98%–114.77%, 83.28%–95.37%, 112.95%–118.06%, 88.08%–120.31%, and 114.40%–116.96%, respectively. Overall, the extraction recoveries and matrix effects were within acceptable ranges, meeting the necessary requirements for analyzing biological samples without compromising the accuracy and reproducibility of the analytical method.

**TABLE 3:** Extraction recovery and matrix effect of icariin, icariside Ⅰ, icariside Ⅱ, baohuoside Ⅱ, wushanicaritin, icaritin, and desmethylicaritin from mouse urine samples (Mean ± SD, *n* = 5).

Analyte	Concentration (ng·mL^−1^)	Extraction recoveries (%)	Matrix effect (%)
Icariin	0.5 (LQC)	92.24	89.50
	20 (MQC)	82.21	84.74
	600(HQC)	92.32	87.10
Icariside Ⅰ	0.5 (LQC)	91.46	98.47
	20 (MQC)	86.07	108.14
	600(HQC)	78.04	106.78
Icariside Ⅱ	0.5 (LQC)	106.04	114.77
	20 (MQC)	96.81	108.98
	600(HQC)	108.71	112.22
Baohuoside Ⅱ	0.5 (LQC)	91.23	94.91
	20 (MQC)	92.02	95.37
	600(HQC)	83.48	83.28
Wushanicaritin	0.5 (LQC)	110.16	116.61
	20 (MQC)	101.56	112.95
	600(HQC)	100.18	118.06
Icaritin	0.5 (LQC)	118.54	120.31
	20 (MQC)	113.77	94.70
	600(HQC)	104.13	88.08
Desmethylicaritin	0.5 (LQC)	110.97	116.96
	20 (MQC)	104.04	115.42
	600(HQC)	99.70	114.40

#### 3.1.5 Stability

The short-term stability at room temperature (25°C) for 6 h, the long-term stability at −20°C for 7 days, the stability after three repeated freeze-thaw cycles at −20°C, and the post-preparation stability in the autosampler (4°C) for 24 h of the standard urine samples at low, medium, and high quality control concentrations (0.5, 20, and 600 ng·mL^−1^, *n* = 5) are detailed in Table 4. The findings revealed that all relative errors (RE) fell within the range of ±15%, suggesting that the stability of analytes in mouse urine met the analytical criteria for biological samples.

**TABLE 4:** Stability of icariin, icariside Ⅰ, icariside Ⅱ, baohuoside Ⅱ, wushanicaritin, icaritin, and desmethylicaritin in mouse urine under various storage conditions (Mean ± SD, *n* = 5).

Analyte	Conc. (ng·mL^−1^)	Short-term matrix stability (25°C, 6 h)		The autosampler stability (4°C, 24 h)		Long-term matrix stability (−20°C, 7 days)		Freeze and thaw stability (−20°C, 3 cycle)	
		Measured (ng·mL^−1^)	RE (%)	Measured (ng·mL^−1^)	RE (%)	Measured (ng·mL^−1^)	RE (%)	Measured (ng·mL^−1^)	RE (%)
Icariin	0.5 (LQC)	0.51 ± 0.04	2.00	0.45 ± 0.02	−10.00	0.49 ± 0.04	−2.00	0.48 ± 0.05	−4.00
	20 (MQC)	19.65 ± 2.03	−1.75	20.33 ± 1.27	1.65	20.99 ± 0.61	4.95	19.08 ± 1.55	−4.60
	600(HQC)	664.96 ± 22.38	10.83	642.07 ± 37.36	7.01	636.33 ± 25.78	6.06	616.06 ± 54.69	2.68
Icariside Ⅰ	0.5 (LQC)	0.49 ± 0.05	−2.00	0.47 ± 0.04	−6.00	0.46 ± 0.02	−8.00	0.45 ± 0.01	−10.00
	20 (MQC)	20.55 ± 1.51	2.75	19.64 ± 2.06	−1.80	21.52 ± 1.53	7.60	19.45 ± 1.58	−2.75
	600(HQC)	671.37 ± 9.15	11.90	571.70 ± 26.72	−4.72	618.32 ± 53.3	3.05	564.20 ± 43.33	−5.97
Icariside Ⅱ	0.5 (LQC)	0.52 ± 0.03	4.00	0.51 ± 0.03	2.00	0.46 ± 0.02	−8.00	0.51 ± 0.03	2.00
	20 (MQC)	18.87 ± 1.55	−5.65	20.27 ± 1.59	1.35	21.28 ± 0.90	6.40	21.10 ± 0.79	5.50
	600(HQC)	541.40 ± 35.21	−9.77	631.98 ± 47.56	5.33	598.61 ± 37.95	−0.23	597.60 ± 38.25	−0.40
Baohuoside Ⅱ	0.5 (LQC)	0.51 ± 0.02	2.00	0.51 ± 0.04	2.00	0.54 ± 0.01	8.00	0.52 ± 0.04	4.00
	20 (MQC)	18.24 ± 1.95	−8.80	19.85 ± 2.39	−0.75	21.78 ± 1.17	8.90	21.39 ± 1.27	6.95
	600(HQC)	628.03 ± 65.49	4.67	563.40 ± 60.50	−6.10	643.32 ± 22.49	7.22	680.92 ± 4.97	13.49
Wushanicaritin	0.5 (LQC)	0.52 ± 0.04	4.00	0.48 ± 0.03	−4.00	0.51 ± 0.04	2.00	0.48 ± 0.04	−4.00
	20 (MQC)	19.26 ± 1.19	−3.70	19.15 ± 0.97	−4.25	20.43 ± 0.27	2.15	18.97 ± 1.00	−5.15
	600(HQC)	588.61 ± 24.12	−1.90	590.56 ± 17.75	−1.57	558.45 ± 35.94	−6.92	555.37 ± 38.24	−7.44
Icaritin	0.5 (LQC)	0.47 ± 0.02	−6.00	0.55 ± 0.01	10.00	0.53 ± 0.03	6.00	0.52 ± 0.04	4.00
	20 (MQC)	19.45 ± 0.88	−2.75	18.37 ± 1.61	−8.15	17.47 ± 0.25	−12.65	17.57 ± 0.44	−12.15
	600(HQC)	532.25 ± 19.13	−11.29	535.76 ± 30.82	−10.71	521.83 ± 6.80	−13.03	537.56 ± 18.15	−10.41
Desmethylicaritin	0.5 (LQC)	0.48 ± 0.05	−4.00	0.48 ± 0.03	−4.00	0.46 ± 0.03	−8.00	0.49 ± 0.06	−2.00
	20 (MQC)	20.61 ± 2.51	3.05	20.75 ± 1.39	3.75	19.68 ± 2.05	−1.60	19.73 ± 2.08	−1.35
	600(HQC)	643.53 ± 22.25	7.26	576.38 ± 61.15	−3.94	574.96 ± 37.91	−4.17	526.64 ± 17.32	−12.23

#### 3.1.6 Dilution integrity

To ensure precise quantification, it was essential to dilute urine samples containing high concentrations of icariside Ⅱ, icaritin, and desmethylicaritin that exceeded the upper limit of the standard curve range before processing. Thus, the dilution integrity of icariside II, icaritin, and desmethylicaritin in mouse urine was assessed. As demonstrated in Table 5, the precision and accuracy of the urine samples remained within ±15% after a 20-fold dilution. This indicated that the samples continued to satisfy the experimental criteria for quantification after undergoing a 20-fold dilution, even when their concentrations surpassed the upper limit of the standard curve range.

**TABLE 5:** 20-fold dilution reliability for assay of icariside II, icaritin, and desmethylicaritin in mouse urine (Mean ± SD, *n* = 5).

Analyte	Concentration (ng·mL^−1^)		RSD (%)	RE (%)
	Expected	Measured		
Icariside Ⅱ	600	662.21 ± 16.17	2.44	10.37
Icaritin		527.81 ± 14.50	2.75	−12.03
Desmethylicaritin		596.84 ± 26.47	4.44	−0.53

### 3.2 Application to urine sample testing

**FIGURE 3:**
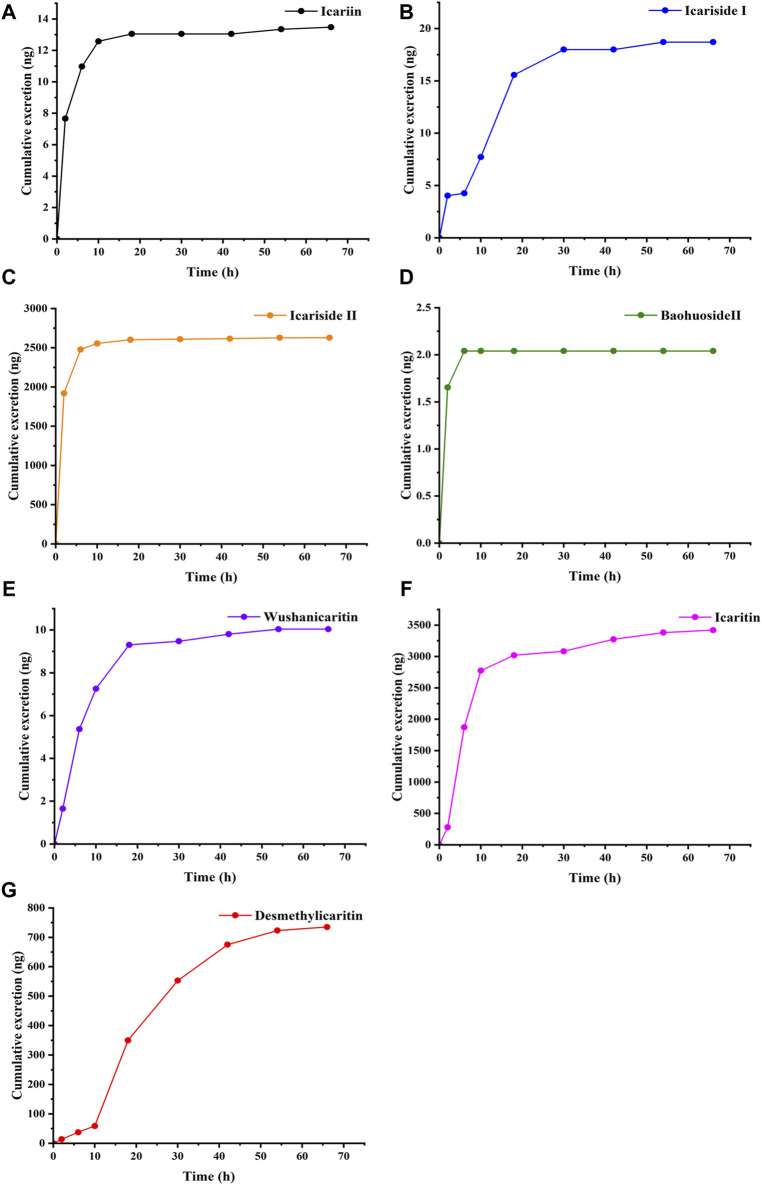
Cumulative excretion of **(A)** Icariin, **(B)** Icariside Ⅰ, **(C)** Icariside Ⅱ, **(D)** Baohuoside Ⅱ, **(E)** Wushanicaritin, **(F)** Icaritin, and **(G)** Desmethylicaritin in mouse urine after intragastric administration of icariin suspension at a dosage of 150 mg·kg^−1^ (*n* = 1).

Following the intragastric administration of an icariin suspension at a dose of 150 mg·kg^−1^ to the C57 mouse, the urinary cumulative excretion-time curves of icariin and its primary metabolites are illustrated in Figure 3. The results of the study indicated that after intragastric administration of icariin, the excretion process of icariin, icariin, icariside Ⅰ, icariside Ⅱ, baohuoside Ⅱ, wushanicaritin, icaritin, and desmethylicaritin commenced within 0–4 h. The cumulative urinary excretion at 72 h was measured as follows: 13.48, 18.70, 2,627.51, 2.04, 10.04, 3,420.44, and 735.13 ng, respectively.

## 4 Discussion

The investigation of metabolites and metabolic processes *in vivo* of traditional Chinese medicines offers substantial evidence for exploring the pharmacodynamic substance foundation of traditional Chinese medicines, thereby promoting their modernization (Wang et al., 2023). After the oral administration of traditional Chinese medicines, their components are absorbed into the bloodstream through the gastrointestinal tract and predominantly undergo phase I and phase II metabolic reactions within the body (Song et al., 2018). During phase I reactions, flavonoids undergo facile oxidation and reduction processes (Huang et al., 2022). Flavonoids exhibit lower polarity and are subject to phase II reactions upon entering the body. They form bonds with sulfuric acid, glucuronic acid, and methyl groups to enhance their polarity, thereby aiding in their elimination from the organism (Yang et al., 2015). The metabolism of icariin in C57 mouse blood was analyzed using the UPLC-MS/MS technique in a prior study conducted in our laboratory (Liu et al., 2023). However, the analysis of icariin and its associated metabolites in biological samples has not been comprehensive. Given that the excretion route effectively provides insights into the mechanism of action of a drug, it is necessary to determine the metabolite profiles and metabolic pathways of icariin in mouse urine (Wang et al., 2021).

Due to complex metabolic pathways, considerable background noise interference, and the limitations associated with the instrumentation used, detecting drugs and their corresponding metabolites *in vivo* poses a significant challenge (Xing et al., 2017). Based on the predicted urinary chemical components after oral administration of icariin, this study aimed to establish a UPLC-MS/MS method for the simultaneous quantitative determination of icariin and its related metabolites in mouse urine. The commonly used chromatographic column, Shiseido CAPCELL PAK MGⅡ C18 (2.0 mm × 100 mm, 3.0 μm), could achieve better retention and separation of small polar compounds. A 0.1% formic acid aqueous solution (A) and a 0.1% formic acid acetonitrile solution (B) were selected as the mobile phases in this study. Formic acid was chosen to acidify the solution to suppress ionization, increase the recovery of icariin and its associated metabolites in the extract, reduce the interference caused by endogenous components in biological samples, improve the sensitivity of instrumental detection, obtain more comprehensive metabolite information, and guarantee optimal peak shape (Cheng et al., 2007; Wang et al., 2022). Pretreatment steps, including purification, extraction, and concentration, were essential to address the significant matrix effect observed in urine samples and the low concentrations (ng‧mL^−1^) of certain analytes to be measured (Anumol and Snyder, 2015). In this study, the sample pretreatment method for biological samples was optimized. After precipitating the protein with either methanol or acetonitrile as the protein precipitator, the urine samples, however, contained endogenous interferences that led to elevated column pressure, distortion of peak shape, and reduced response values for the target analytes. The results showed that the simple liquid-liquid extraction technique for sample pretreatment could overcome these issues and fulfill the assay requirements. In the analysis of biological samples, internal standards are typically required to correct for specific sample deviations in losses resulting from processing and chromatography. In the present study, propranolol was chosen as the internal standard due to its physical and chemical properties and chromatographic behavior compared favorably with icariin (Cheng et al., 2007).

Using the aforementioned conditions for sample preparation and chromatography, icariin and its six metabolites, including icariside Ⅰ, icariside Ⅱ, baohuoside Ⅱ, wushanicaritin, icaritin, and desmethylicaritin, could be rapidly extracted from mouse urine and effectively separated from other endogenous interferences. To study the pharmacokinetics of icariin in rats, Shen et al. employed LC-MS/MS to identify icariin, icariside Ⅰ, and icariside Ⅱ, icaritin, and desmethylicaritin in rat serum. The analysis lasted 12 min, with a detection limit of 0.78 nM, and linearity ranges of 0.78–12.5, 0.78–100, 0.78–100, 0.78–12.5, and 0.78–12.5 nM, respectively (Shen et al., 2009). Besides, Yu et al. used LC-MS/MS to identify epimedin A, epimedin B, epimedin C, and icariin in rat urine, with a LLOQ of 1 ng‧mL^−1^ and a linearity range of 1–500 ng‧mL^−1^ (Yu et al., 2016). Our proposed method was a rapid one for the determination of the icariin and its six metabolites in less than 7 min. It offered a shorter analytical time and a lower LLOQ compared to the methods mentioned above. The method established conformed to the “9012 Guidelines for Quantitative Bioanalytical Method Validation” outlined in the Chinese Pharmacopoeia (Chinese Pharmacopoeia Commission, 2020b). In the urine excretion experiment, following the intragastric administration of an icariin suspension at a dosage of 150 mg·kg^-1^ to the C57 mouse, icariin and its related metabolites were detected in the urine. The assay results showed that after oral administration of icariin, the excretion process of icariin, icariside Ⅰ, icariside Ⅱ, baohuoside Ⅱ, wushanicaritin, icaritin, and desmethylicaritin started from 0 to 4 h. The cumulative urinary excretion at 72 h was 13.48, 18.70, 2,627.51, 2.04, 10.04, 3,420.44, and 735.13 ng, respectively. The findings suggested that only a minor proportion of the administered dosage of icariin and its metabolites were excreted via urine, indicating that the renal route was not the primary elimination pathway for the prodrug icariin. Icariside Ⅱ, icaritin, and desmethylicaritin were the metabolites with higher abundance in urine. It was shown that the established method was suitable for investigating the urinary excretion of icariin.

Previous studies indicated that the oral bioavailability of Epimedium flavonoids was very low (Lee et al., 2013). After oral administration, 91.2% of icariin was converted to icariside II, the predominant form in rat plasma. Following intravenous administration, only 0.4% of icariin was converted to icariside II, highlighting the significant contribution of intestinal microbiota (intestinal flora) in metabolizing orally administered icariin (Cheng et al., 2015). Chen Y et al. examined the influence of small intestinal hydrolases on the absorption of Epimedium flavonoids and found that the flavonoids were hydrolyzed into secondary glycosides in the small intestine before absorption (Chen et al., 2011). The metabolism of icariin in the human intestine was similar to that reported in animal models. Research conducted by Wu et al. indicated that the majority of icariin was hydrolyzed by lactase phlorizin hydrolase and microbiota β-glucosidase into metabolites including icariside II (primarily), icaritin, and desmethylicaritin rapidly prior to absorption in the human intestinal tract, with icariside I being an exception. Furthermore, due to personal variations in the host’s microflora, the metabolism of icariin exhibited interindividual differences (Wu et al., 2016). Wang et al. simultaneously used positive and negative ion modes for UHPLC/Q-TOF-MS/MS analysis and detection. The study results indicated that the major metabolites in different biological samples (feces, urine, bile, and plasma) varied. Most of the detectable metabolites were found in the feces. It was initially hypothesized that icariin was hydrolyzed to icariside Ⅰ, icariside Ⅱ, and icaritin in the small intestine of the rats. Subsequently, it was absorbed into the bloodstream. The C7-OH of the absorbed icariside Ⅱ was then glucuronidated in the liver and transformed into bile or released into the bloodstream. Finally, it was excreted in the feces and urine (Wang et al., 2019). These studies have confirmed that icariin is seldom absorbed and eliminated as a prodrug *in vivo*. Instead, the primary route of elimination was through metabolic pathways. The results of this experiment hold great significance for improving the pharmacokinetic studies of icariin and understanding its biotransformation pattern *in vivo*. Meanwhile, the analytical method and excretion experiment results provide a foundation for the study of icariin and its metabolites in APP/PS1 transgenic mice, effectively supporting research on these mice.

## 5 Conclusion

Based on the preliminary pharmacokinetic studies, this section of the experimental research established a sensitive and effective UPLC-MS/MS method for quantifying the concentrations of icariin and its associated metabolites, including icariside Ⅰ, icariside Ⅱ, baohuoside Ⅱ, wushanicaritin, icaritin, and desmethylicaritin in the urine samples obtained from C57 mice. Bioanalytical method investigations were conducted, encompassing evaluations of selectivity, precision and accuracy, extraction recovery and matrix effect, stability, and dilution integrity. These assessments were found to align with the necessary criteria for sample analysis. The results have showed the accuracy and reproducibility of this method, indicating its suitability for the simultaneous determination of icariin and its associated metabolites in mouse urine. Moreover, the method can be applied to analyze urine samples containing multiple drugs. This sets the groundwork for further research on the metabolic processes and *in vivo* mechanisms of action of icariin, aiming to enhance the pharmacokinetic investigations of icariin.

## Data Availability

The original contributions presented in the study are included in the article/supplementary material, further inquiries can be directed to the corresponding author.
